# Characterization of Piperacillin/Tazobactam-Resistant *Klebsiella oxytoca* Recovered from a Nosocomial Outbreak

**DOI:** 10.1371/journal.pone.0142366

**Published:** 2015-11-05

**Authors:** Ai Fujita, Kouji Kimura, Satoru Yokoyama, Wanchun Jin, Jun-ichi Wachino, Keiko Yamada, Hiroyuki Suematsu, Yuka Yamagishi, Hiroshige Mikamo, Yoshichika Arakawa

**Affiliations:** 1 Department of Bacteriology, Nagoya University Graduate School of Medicine, 65 Tsurumai-cho, Showa-ku, Nagoya 466-8550, Japan; 2 Department of Clinical Infectious Diseases, Aichi Medical University Graduate School of Medicine, 1-1 Yazakokarimata, Nagakute, Aichi 480-1195, Japan; The University of Hong Kong, HONG KONG

## Abstract

We characterized 12 clinical isolates of *Klebsiella oxytoca* with the extended-spectrum β-lactamase (ESBL) phenotype (high minimum inhibitory concentration [MIC] values of ceftriaxone) recovered over 9 months at a university hospital in Japan. To determine the clonality of the isolates, we used pulsed-field gel electrophoresis (PFGE), multi-locus sequence typing (MLST), and PCR analyses to detect *bla*_RBI_, which encodes the β-lactamase RbiA, OXY-2-4 with overproduce-type promoter. Moreover, we performed the isoelectric focusing (IEF) of β-lactamases, and the determination of the MICs of β-lactams including piperacillin/tazobactam for 12 clinical isolates and *E*. *coli* HB101 with pKOB23, which contains *bla*_RBI_, by the agar dilution method. Finally, we performed the initial screening and phenotypic confirmatory tests for ESBLs. Each of the 12 clinical isolates had an identical PFGE pulsotype and MLST sequence type (ST9). All 12 clinical isolates harbored identical *bla*_RBI_. The IEF revealed that the clinical isolate produced only one β-lactamase. *E*. *coli* HB101 (pKOB23) and all 12 isolates demonstrated equally resistance to piperacillin/tazobactam (MICs, >128 μg/ml). The phenotypic confirmatory test after the initial screening test for ESBLs can discriminate β-lactamase RbiA-producing *K*. *oxytoca* from β-lactamase CTX-M-producing *K*. *oxytoca*. Twelve clinical isolates of *K*. *oxytoca*, which were recovered from an outbreak at one university hospital, had identical genotypes and produced β-lactamase RbiA that conferred resistance to piperacillin/tazobactam. In order to detect *K*. *oxytoca* isolates that produce RbiA to promote research concerning β-lactamase RbiA-producing *K*. *oxytoca*, the phenotypic confirmatory test after the initial screening test for ESBLs would be useful.

## Introduction

*Klebsiella oxytoca*, a member of the *Enterobacteriaceae*, is a Gram-negative opportunistic pathogen that causes pneumonia, bacteraemia, urinary tract infections, and enterocolitis [1, 2]. The chromosome of *K*. *oxytoca* typically encodes a class A β-lactamase designated OXY (previously called K1 or KOXY) [3]. *K*. *oxytoca* strains, which overproduce OXY due to a point mutation in the promoter region that confers resistance to broad-spectrum β-lactams, aztreonam (ATM) as well as to β-lactamase inhibitors, were reported approximately 24 years ago [4–10]. There are recent reports of *K*. *oxytoca* isolates that produce plasmid-encoded β-lactamases, including extended-spectrum β-lactamases (ESBLs) and carbapenemases [11–14]. A recent nosocomial outbreak caused by *K*. *pneumoniae* carbapenemase (KPC)-producing *K*. *oxytoca* isolates was reported as well [15, 16].

Although research has focused on carbapenemase-producing *K*. *oxytoca* isolates, *K*. *oxytoca* strains that produce ESBLs or overproduce OXY must not be overlooked. The β-lactamase OXY group comprises the OXY-1, OXY-2, OXY-3, OXY-4, OXY-5 and OXY-6 subgroups [17–19]. Strains that overproduce the chromosomally encoded β-lactamase OXY are resistant to all β-lactamase inhibitors [9, 20, 21]. For example, we earlier reported that, in Japan, a variant of OXY with an overproduce-type promoter that drives the expression of the β-lactamase, RbiA (accession number D84548, OXY-2-4), shows resistance to β-lactamase inhibitors [20]. The combination of piperacillin, a penicillin antibiotic, and tazobactam, a β-lactamase inhibitor (TZP), is now widely used in Japan, because most *Klebsiella* species are susceptible to TZP [22].

We experienced an outbreak caused by *K*. *oxytoca* with the ESBL phenotype (high minimum inhibitory concentration [MIC] value of ceftriaxone [CRO]) at a university hospital in Japan. Here, we report the characterization of clinical isolates of *K*. *oxytoca* derived from this outbreak over a period of 9 months.

## Materials and Methods

### Ethics statement

We used clinical information concerning clinical isolates analyzed in this study. All the clinical information was approved by the ethical committee of the Aichi Medical University Graduate School of Medicine.

### Clinical information

This outbreak was declared in June 2009 and containment of the outbreak was declared in December 2010. The outbreak has been ended by enforcing strict hand hygiene, strict contact precaution and promotion of antimicrobial stewardship. *K*. *oxytoca* clinical isolates NUBL-1520, 1521, 1522, 1523, 1524, 1525, 1526, 1527, 1528, 1529, 1530, and 1531 were recovered from 8 different patients at one university hospital in Aichi, Japan from 2009 June to 2010 February (Table 1). All the patients were inpatients, admitted at the identical ward of neurosurgery, for various operations. The outcomes of all the patients were survival or change of hospital.

**Table 1:** Clinical information concerning *Klebsiella oxytoca* NUBL1520-1531.

Clinical isolates	Patient	Age (yr.)	Sex	Isolation date (mo./day/yr.)	Specimen	Underlying disease	Judgment of infection	Treatment for infection
NUBL1520	Patient A	72	F	6/5/2009	Sputum	Hypertension, diabetes	Colonization	N.A.
NUBL1521	Patient A			6/9/2009	IHC		Colonization	N.A.
NUBL1522	Patient B	56	M	7/4/2009	Urine	Hypertension	Colonization	N.A.
NUBL1523	Patient C	75	F	7/22/2009	Urine	Diabetes	Colonization	N.A.
NUBL1524	Patient C			7/22/2009	Sputum		Colonization	N.A.
NUBL1525	Patient B			8/17/2009	Sputum		Colonization	N.A.
NUBL1526	Patient D	46	M	9/25/2009	Pus	N.A.	PSSTI	MEPM
NUBL1527	Patient E	56	M	9/24/2009	Sputum	N.A.	Colonization	N.A.
NUBL1528	Patient F	37	M	9/28/2009	Sputum	N.A.	Pneumonia	DRPM
NUBL1529	Patient G	17	F	9/28/2009	Sputum	N.A.	Pneumonia	DRPM
NUBL1530	Patient H	73	F	2/15/2010	Urine	Hypertension, diabetes	Colonization	N.A.
NUBL1531	Patient H			2/15/2010	Sputum		Pneumonia	MEPM

Abbreviations: F, female; M, male; IHC, intravenous hyperalimentation catheter; N.A., not applicable; PSSTI, postoperative skin and soft-tissue infection; MEPM, meropenem; DRPM, doripenem.

### Clinical isolates

NUBL-1521 was isolated from an intravenous hyperalimentation catheter, NUBL-1522, 1523 and 1530 were isolated from urine samples and NUBL-1526 was isolated from pus. All other isolates were recovered from sputum (Table 1).

### Plasmid vectors

The plasmid pKOB23 [20] harbors the *bla*_RBI_ gene of *K*. *oxytoca* SB23, which is carried by the pMK16 cloning vector.

### Reagents

Ampicillin (AMP) and cefotaxime (CTX) were purchased from Wako Pure Chemical Industries, LTD. Piperacillin (PIP) and tazobactam were purchased from LKT Laboratories, Inc. Imipenem (IPM) was purchased from Ark Pharm. The disks used for Screening and Confirmatory Tests for ESBLs contained the antibiotics as follows: cefpodoxime (CPD), ATM, CRO, ceftazidime (CAZ), and CTX disks were purchased from Becton, Dickinson and Company. Clavulanic acid (CLA) was purchased from Wako Pure Chemical Industries, LTD.

### Pulsed-field gel electrophoresis (PFGE)

Plugs were prepared using suspensions of clinical isolates with an optical density of 0.8; these plugs had treated with 2 mg/ml of lysozyme solution at 37°C for 6 h and 1 mg/ml of proteinase K solution at 55°C for 8 h. The digested plugs were incubated with XbaI (Takara). We performed PFGE for 24 h using a CHEF-DR III System (BioRad). Gels were stained with 0.5 μg/ml of ethidium bromide for 1 h.

### Multi-locus sequence typing (MLST)

We performed MLST analysis of the *K*. *oxytoca* isolates as described previously [23]. We isolated chromosomal DNA using a Wizard Genomic DNA Purification Kit (Promega). The seven housekeeping genes were amplified using PCR with the high-fidelity PrimeSTAR HS DNA polymerase (Takara). Nucleotide sequences were determined using an Applied Biosystems 3130xl Genetic Analyzer or an Applied Biosystems 3730xl DNA Analyzer and BigDye Terminator V3.1. We determined the sequence type (ST) using the *K*. *oxytoca* MLST website (http://pubmlst.org/koxytoca/).

### PCR detection of β-lactamase RbiA gene

We performed the chromosomal DNA isolation from *K*. *oxytoca* NUBL-1520, 1521, 1522, 1523, 1524, 1525, 1526, 1527, 1528, 1529, 1530 and 1531, using Wizard Genomic DNA Purification Kit (Promega). We performed PCR reaction using the purified chromosomal DNA as templates, high fidelity DNA polymerase, PrimeSTAR HS DNA polymerase (Takara), and previously described primers, OXY-383 and OXY-S [7]. The nucleotide sequences of the amplicons were determined as described above.

### Isoelectric focusing (IEF) of β-lactamases

To extract β-lactamases from the clinical isolate NUBL-1520, we performed a freeze-thaw procedure [24] and subjected the resulting supernatant to IEF using an Invitrogen system. IEF was conducted for 1 h at 100 V, 2 h at 200 V and 30 min at 500 V. The β-lactamase in the gel was detected using 0.05% nitrocefin solution [25].

### Determination of MICs

The MICs of AMP, PIP, TZP, CTX, and IPM were determined according to the guidelines of the Clinical and Laboratory Standards Institute (CLSI) using the agar dilution method [26]. *E*. *coli* ATCC 25922 and *E*. *coli* ATCC 35218 strains served as controls.

### Screening and Confirmatory Tests for ESBLs

We performed the disk diffusion method recommended by CLSI called the Screening and Confirmatory Tests for ESBLs [26]. In the Initial Screen Test, we used CPD, and CRO, and ATM disks. For *K*. *oxytoca*, the breakpoints of the CPD zone, CRO zone, and ATM zone are ≤17 mm, ≤25 mm, and ≤27 mm, respectively. According to the CLSI, “Zones above may indicate ESBL production.” In Phenotypic Confirmatory Test, we used CAZ, CAZ-CLA, CTX, and CTX-CLA disks. Confirmatory testing requires the use of both CAZ and CTX, alone and in combination with CLA. According to the CLSI, “a ≥5 mm increase in the zone diameter for either antimicrobial agent tested in combination with CLA vs. its zone when tested alone = ESBL.” We used NUBL-793 and 810, which have been already confirmed as β-lactamase CTX-M-producing *K*. *oxytoca*.

## Results

### MICs at a clinical setting

The MICs for the 12 clinical isolates of *K*. *oxytoca* determined at a microbiological laboratory of a university hospital are shown in Table 2. All isolates were resistant to cefazolin, cefotiam, and CRO. At first, the laboratory technicians missed the high MIC values of sultamicillin and cefoperazone/sulbactam. Therefore, they suspected these clinical isolates as ESBL-producing *K*. *oxytoca*, because of their high MIC values of CRO.

**Table 2:** MIC values of *K*. *oxytoca* NUBL1520-1531 determined at a microbiological laboratory of a university hospital.

Clinical isolates	MIC [μg/ml]											
	SBTPC	CFZ	CTM	CFP/SUL	CAZ	CRO	CZOP	CFPN	IPM	LVX	FOF	SXT
NUBL1520	>32	>16	16	>16/16	≤0.5	32	32	≤1	≤0.5	4	128	≤0.25/4.75
NUBL1521	>32	>16	>32	>16/16	2	>32	32	2	≤0.5	>4	>128	≤0.25/4.75
NUBL1522	>32	>16	32	>16/16	2	32	8	≤1	≤0.5	>4	128	≤0.25/4.75
NUBL1523	>32	>16	16	>16/16	1	>32	>32	≤1	≤0.5	>4	>128	≤0.25/4.75
NUBL1524	>32	>16	16	>16/16	1	>32	>32	≤1	≤0.5	>4	>128	≤0.25/4.75
NUBL1525	>32	>16	16	>16/16	1	32	>32	≤1	≤0.5	>4	>128	≤0.25/4.75
NUBL1526	>32	>16	32	>16/16	1	16	>32	≤1	≤0.5	>4	>128	≤0.25/4.75
NUBL1527	>32	>16	>32	>16/16	1	>32	>32	≤1	≤0.5	>4	128	≤0.25/4.75
NUBL1528	>32	>16	>32	>16/16	4	>32	32	>8	≤0.5	4	>128	≤0.25/4.75
NUBL1529	>32	>16	>32	>16/16	1	>32	>32	≤1	≤0.5	>4	128	≤0.25/4.75
NUBL1530	>32	>16	32	>16/16	1	16	≤1	≤1	≤0.5	>4	>128	≤0.25/4.75
NUBL1531	>32	>16	32	>16/16	1	16	≤1	≤1	≤0.5	>4	>128	≤0.25/4.75

Abbreviations: MIC, minimum inhibitory concentration; SBTPC, sultamicillin; CFZ, cefazolin; CTM, cefotiam; CFP, cefoperazone; SUL, sulbactam; CAZ, ceftazidime; CRO, ceftriaxone; CZOP, cefozopran; CFPN, cefcapene; IPM, imipenem; LVX, levofloxacin; FOF, fosfomycin; SXT, trimethoprim-sulfamethoxazole.

### PFGE analysis

**Fig 1:**
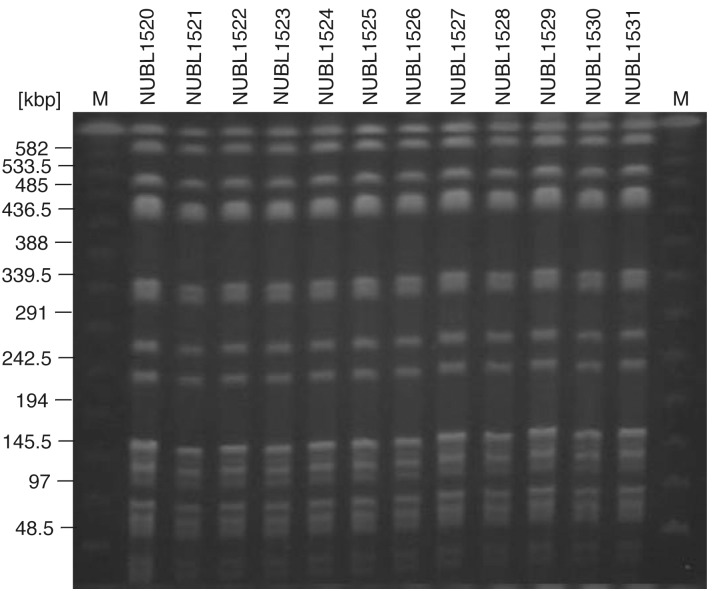
Pulsed-field gel electrophoresis (PFGE) analysis of 12 clinical isolates of *K*. *oxytoca*. M, size markers.

All clinical isolates exhibited the identical pulsotype (Fig 1), suggesting that they possessed identical genotypes, which indicates that the outbreak was caused by the same clinical isolate.

### MLST

All 12 clinical isolates were ST9, indicating that they possessed the identical genotype.

### PCR detection of the β-lactamase RbiA gene

Because of the high MIC values of sultamicillin and cefoperazone/sulbactam and our previous findings that the β-lactamase RbiA confers resistance to β-lactamase inhibitors upon *K*. *oxytoca* [20], we performed PCR and nucleotide sequence analyses to detect *bla*_RBI_ and found the *bla*_RBI_ sequences of all isolates were identical (Accession Number D84548), including the –35 and –10 regions, the Shine-Dalgarno sequence, and the coding region. This supports the clonal origin of the 12 clinical isolates.

### IEF analysis of β-lactamases

**Fig 2:**
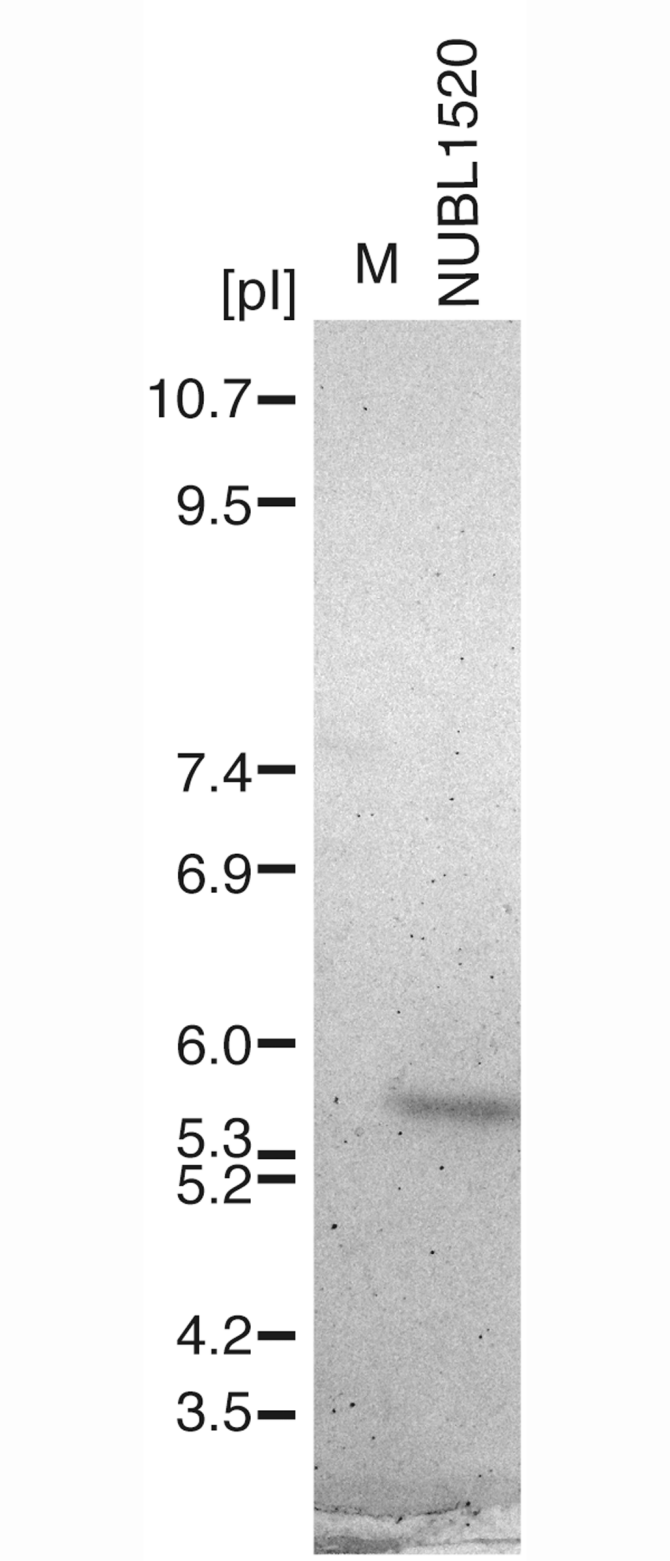
Isoelectric focusing (IEF) of the β-lactamase of clinical isolate NUBL1520. M, pI markers.

To determine the number of β-lactamases produced by the clinical isolates, we performed IEF (Fig 2). A single band was detected at pH 5.6, suggesting that NUBL1520 produces ‘only one’ β-lactamase and supporting that only one β-lactamase produced by NUBL1520 is β-lactamase RbiA [20, 27].

### Determination of MICs

Although the wide use of TZP started recently in Japan and there are a few reports concerning TZP resistant *K*. *oxytoca* that produce OXY-2 type β-lactamase [12, 28], it remained to be determined whether *K*. *oxytoca* strains that produce RbiA are resistant to TZP. Therefore, we determined the MICs of five β-lactams, including TZP, for the 12 clinical isolates and *E*. *coli* HB101 (pKOB23) that harbors *bla*_RBI_. All isolates were resistant to AMP, PIP and TZP, exhibited intermediate or resistance to CTX and were susceptible to IPM (Table 3). *E*. *coli* HB101 (pKOB23) and all 12 isolates demonstrated equally resistance to TZP (MICs, >128 μg/ml), and *E*. *coli* HB101 (pMK16) showed susceptibility to TZP (MIC, 1 μg/ml), suggesting that β-lactamase RbiA confers to resistance to TZP in 12 clinical isolates of *K*. *oxytoca*.

**Table 3:** MIC values of β-lactams for *K*. *oxytoca* NUBL1520-1531, *E*. *coli* HB101 (pKOB23), and *E*. *coli* HB101 (pMK16) determined using the agar dilution method.

Clinical isolates, Strains	MICs [μg/ml]				
	AMP	PIP	TZP	CTX	IPM
NUBL 1520	>128	>128	>128	8	0.5
NUBL 1521	>128	>128	>128	16	1
NUBL 1522	>128	>128	>128	4	0.25
NUBL 1523	>128	>128	>128	4	0.12
NUBL 1524	>128	>128	>128	4	0.12
NUBL 1525	>128	>128	>128	2	0.12
NUBL 1526	>128	>128	>128	2	0.12
NUBL 1527	>128	>128	>128	4	0.5
NUBL 1528	>128	>128	>128	4	0.25
NUBL 1529	>128	>128	>128	4	0.25
NUBL 1530	>128	>128	>128	2	0.12
NUBL 1531	>128	>128	>128	4	0.25
*E*. *coli* HB101 (pKOB23)	>128	>128	>128	8	0.25
*E*. *coli* HB101 (pMK16)	8	4	1	0.06	0.25

Abbreviations: MIC, minimum inhibitory concentration; AMP, ampicillin; PIP, piperacillin; TZP, piperacillin-tazobactam; CTX, cefotaxime; IPM, imipenem.

### Initial Screening and Confirmatory Tests for ESBLs

Although it was previously reported that many β-lactamase K1-overproducing *K*. *oxytoca* strains show false-positive in ESBL tests [29], no data were available indicating whether the initial screening and confirmatory tests for ESBLs recommended by CLSI detect *K*. *oxytoca* clinical isolates that produce RbiA. Therefore, we tested the clinical isolates along with the control strains *K*. *oxytoca* NUBL793 and NUBL810 that produce CTX-M. In the initial screening test, the diameters of inhibition surrounding the CPD, ATM, and CRO disks in plates containing NUBL793, NUBL810 as well as those of all clinical isolates were less than the cut-off values recommended by CLSI, suggesting that all clinical isolates may produce ESBLs (Table 4). In the phenotypic confirmatory test, NUBL793 and NUBL810 showed an obvious increase (≥5 mm) of the diameters of the CTX-CLA and CTX disks; however, none of the 12 clinical isolates showed the necessary increase of the diameters between CTX-CLA disk and CTX alone disk, and CAZ-CLA disk and CAZ alone disk (Table 5), suggesting that the phenotypic confirmatory test discriminates *K*. *oxytoca* strains that produce RbiA from those that produce CTX-M.

**Table 4:** Initial Screening Tests for ESBLs.

Strains and clinical isolates	CPD 10 μg	ATM 30 μg	CRO 30 μg
NUBL793 (*K*. *oxytoca* CTX-M1)	8	12	10
NUBL810 (*K*. *oxytoca* CTX-M2)	6	20	12
NUBL1520	16	13	15
NUBL1521	8	6	9
NUBL1522	12	7	14
NUBL1523	13	8	15
NUBL1524	13	8	15
NUBL1525	13	8	14
NUBL1526	13	9	15
NUBL1527	14	8	15
NUBL1528	17	17	19
NUBL1529	15	9	16
NUBL1530	13	9	15
NUBL1531	14	10	18

For *K*. *oxytoca*, the breakpoints of the CPD, CRO, and ATM zones are ≤17 mm, ≤25 mm, and ≤27 mm, respectively. According to the CLSI, “Zones above may indicate ESBL production.”

Abbreviations: ESBL, extended-spectrum β-lactamase; CPD, cefpodoxime; ATM, aztreonam; CRO, ceftriaxone.

**Table 5:** Phenotypic Confirmatory Tests for ESBLs.

Clinical isolates, Strains	CAZ 30 μg	CAZ-CLA 30/10 μg	CTX 30 μg	CTX-CLA 30/10 μg
NUBL793 (*K*.*oxytoca* CTX-M1)	26	27	18	23
NUBL810 (*K*. *oxytoca* CTX-M2)	24	30	15	28
NUBL1520	27	27	22	24
NUBL1521	20	21	13	12
NUBL1522	21	20	21	21
NUBL1523	21	21	23	23
NUBL1524	21	21	21	22
NUBL1525	21	21	23	23
NUBL1526	23	23	23	22
NUBL1527	23	24	24	25
NUBL1528	28	30	26	27
NUBL1529	26	24	24	25
NUBL1530	22	22	23	23
NUBL1531	25	26	25	27

Confirmatory testing requires the use of both CAZ and CTX, alone and in combination with CLA. According to the CLSI, “a ≥5 mm increase in the zone diameter for either antimicrobial agent tested in combination with CLA vs. its zone when tested alone = ESBL.”

Abbreviations: ESBL, extended-spectrum β-lactamase; CAZ, ceftazidime; CLA, clavulanic acid; CTX, cefotaxime.

## Discussion

We show here that 12 clinical isolates of *K*. *oxytoca*, which we recovered from an outbreak at one university hospital, had identical genotypes and produced β-lactamase RbiA that conferred resistance to TZP. Moreover, we demonstrated that the phenotypic confirmatory test after the initial screening test for ESBLs recommended by CLSI is useful for discriminating *K*. *oxytoca* clinical isolates that produce RbiA from those that produce CTX-M. It has been reported previously that some ESBL-producing clinical isolates of *Klebsiella* spp. are resistant to TZP [30, 31], and that only 12 of 25 *Enterobacteriaceae* strains producing ESBLs are susceptible to TZP [32]. However, although several studies have examined ESBL-producing clinical isolates [33], the number of reports concerning *K*. *oxytoca* clinical isolates producing β-lactamase RbiA is limited. Therefore, in order to promote research on *K*. *oxytoca* clinical isolates producing β-lactamase RbiA, it is important to discriminate *K*. *oxytoca* clinical isolates that produce RbiA from ESBL-producing *K*. *oxytoca* clinical isolates and to characterize *K*. *oxytoca* clinical isolates producing β-lactamase RbiA.

TZP is often prescribed for patients treated in the hospital studied here (data not shown). It is possible that the outbreak described here was caused by selection by TZP of *K*. *oxytoca* strains that produce RbiA. Moreover, the amount of TZP prescribed in Japan may be increasing in concert with the increase in ESBL-producing *Enterobacteriaceae*. Therefore, it is reasonable to assume that outbreaks similar to that described here will occur again.

It is difficult to readily discriminate between ESBL-producing *K*. *oxytoca* strains and those that produce RbiA because of the ESBL-phenotype (high MIC values of CRO et al.) of the latter. However, we show here that *K*. *oxytoca* clinical isolates that produce RbiA are resistant to TZP. Moreover, in our hands, the confirmatory test after the initial screening test for ESBLs recommended by CLSI were useful for discriminating between the two *K*. *oxytoca* phenotypes. In order to detect *K*. *oxytoca* isolates that produce RbiA to promote research concerning β-lactamase RbiA-producing *K*. *oxytoca*, the phenotypic confirmatory test after the initial screening test for ESBLs would be useful.
